# Simultaneous Bilateral Patellar Tendon Rupture: A Systematic Review

**DOI:** 10.7759/cureus.41512

**Published:** 2023-07-07

**Authors:** André Fernandes, Mariana Rufino, Divakar Hamal, Amr Mousa, Emma Fossett, Kamalpreet S Cheema

**Affiliations:** 1 Trauma and Orthopaedics, York and Scarborough Teaching Hospitals NHS Foundation Trust, York, GBR; 2 Respiratory Medicine, Wirral University Teaching Hospital NHS Foundation Trust (WUTH), Wirral, GBR; 3 Anesthesiology, Hull University Teaching Hospital, Hull, GBR; 4 Surgery, The Hillingdon Hospital NHS Foundation Trust, London, GBR; 5 Trauma and Orthopaedics Surgery, Guy's and St. Thomas' NHS Foundation Trust, London, GBR

**Keywords:** patella tendon, knee trauma surgeon, trauma management, ligamentous knee injury, knee trauma, orthopaedics, bilateral patella tendon rupture, systematic review, bilateral, patellar tendon rupture

## Abstract

The extensor mechanism of the knee can be damaged due to various modes of injury, which, in most cases, will require urgent surgical intervention for repair. Single patellar tendon ruptures are uncommon, but simultaneous bilateral events are even rarer and have been scarcely reviewed in English literature. Research in this area is mainly confined to case series, with some literature reviews but no evidence of more substantial analysis. Therefore, this systematic review was done to analyse the existing literature on bilateral simultaneous patellar tendon ruptures and propose a systematic and standardised approach to diagnosing and managing these injuries.

A systematic review was conducted using the Preferred Reporting Items for Systematic Reviews and Meta-Analysis (PRISMA) guidelines. The search terms included ‘bilateral patellar tendon rupture’, ‘bilateral’, ‘patellar’, ‘tendon’ and ‘rupture’. Three independent reviewers conducted searches in PubMed, OvidSP for Medline, Embase and the Cochrane Library using the same search strategy. The eligibility criteria included studies on bilateral concomitant patellar tendon rupture published in English. Bilateral simultaneous patellar tendon ruptures of traumatic and atraumatic origin in human patients were included. The study types comprised case reports and literature reviews.

The key limitation of this study was the low number of patients covered by the eligible literature. Patellar tendon ruptures are a rare and scarcely documented injury, and there is a need for studies with a high level of evidence, especially regarding surgical treatment choice and methods, as well as post-operative management, which could potentially lead to improved outcomes in the management of this injury.

## Introduction and background

Single patellar tendon ruptures are relatively uncommon, and bilateral patellar tendon ruptures are even rarer and have scarcely been reviewed in the literature, presenting mostly as low-quality evidence with the odd literature review [1]. These studies highlight specific predisposing risk factors for bilateral patellar tendon ruptures, such as hyperuricemia, lupus erythematosus and hyperparathyroidism, which cause microstructural changes, leading to weakened ligament collagen [2]. However, the causes for tendon ruptures in the literature are predominantly categorised as systemic conditions, chronic microtrauma or steroid use, with only a few studies presenting predisposing factors for degenerative changes of the patellar tendon, and explaining the bilateral and simultaneous nature of this injury remains challenging [3].

This systematic review will evaluate the literature on bilateral patellar tendon ruptures by identifying patient predisposing factors, mode and level of injury, investigations, Insall-Salvati ratios, surgical techniques and intra- and post-operative complications. Furthermore, this review aims to answer some key questions: (1) can we optimise patient factors to allow a better management outcome, (2) how can we improve diagnostics to reduce morbidity in this subset of patients and (3) what is the best method of surgical repair for ruptured patellas? After our analysis, we aim to suggest a systematic and standardised approach to managing patients with bilateral patellar tendon ruptures and reducing their incidence.

The knee’s extensor mechanism involves four components: the tibial tubercle, the patella tendon, the patella and the quadriceps tendon. Injury to any of these four components can lead to the disruption of the extensor mechanism. Patellar tendon ruptures are the rarest type of injury among patients with no predisposing factors, affecting less than 0.5% of the population yearly, possibly because a force of almost 17.5 times one’s body weight is required to rupture the patella tendon [4]. Siwek and Rao [5] also found that spontaneous patellar tendon ruptures can often be misdiagnosed as tendinitis due to its fairly indolent and atraumatic course, with 28% misdiagnosed on initial examination.

The clinical presentation of bilateral ruptured patellar tendons varies due to the mechanism of injury, as well as the concordant risk factors, which include a history of pain with or without a history of trauma, loss or limitation of knee extension, knee swelling, a palpable gap in the tendon and a high-riding patella. A plain anterior-posterior (AP) and lateral radiograph of the knee can not only rule out bony injuries but also detect relevant radiographic findings, such as a raised Insall-Salvati ratio (ratio of the patella tendon length to the length of the patella, used to determine patellar height) of >1.2 of both knees without signs of osseous involvement, indicating a patella alta and a compromised patella tendon apparatus. However, a tensile overload of the knee can also lead to an associative tibial tubercle avulsion fracture [6]. A high-riding patella can also be due to an underlying joint effusion [7]. However, it is important to rule out the other three mechanisms of knee extensor disruption before reaching a final diagnosis [8].

## Review

Materials and methods

**Figure 1:**
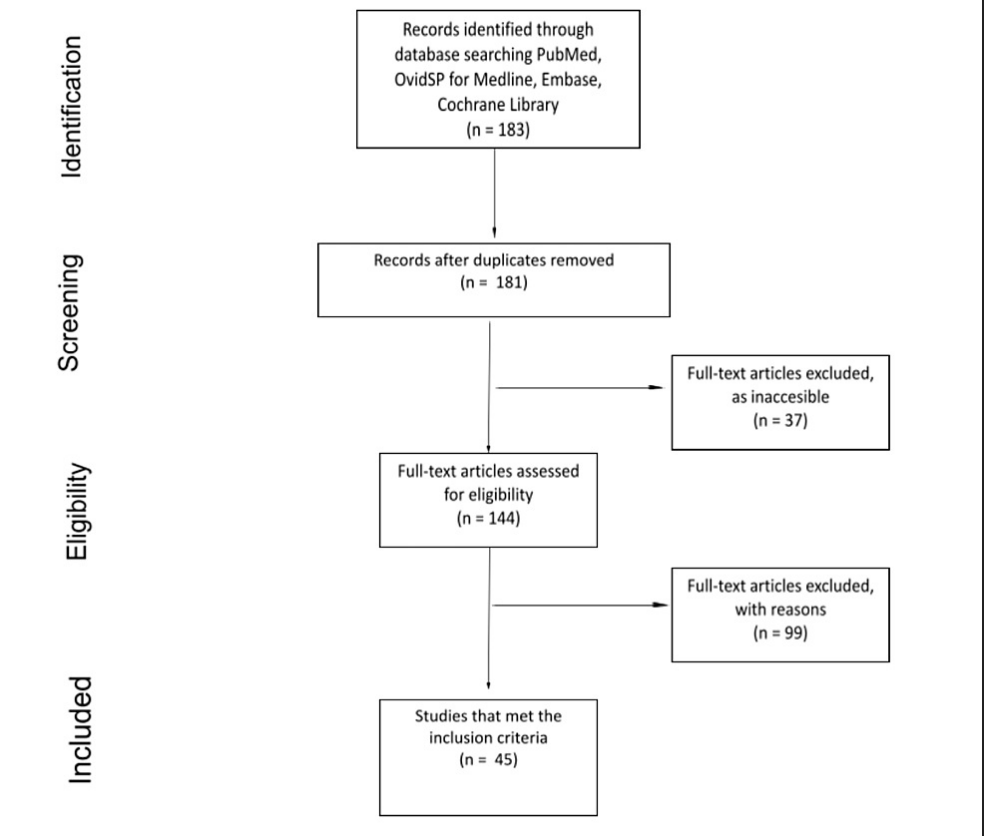
PRISMA literature search methodology. PRISMA: Preferred Reporting Items for Systematic Reviews and Meta-Analysis Copyright Disclaimer under section 107 of the Copyright Act 1976: allowance is made for ‘fair use’ for purposes such as criticism, comment, news reporting, teaching, scholarship, education and research.

A systematic review was conducted using the Preferred Reporting Items for Systematic Reviews and Meta-Analysis (PRISMA) guidelines. The search terms included ‘bilateral patellar tendon rupture’, ‘bilateral’, ‘patellar’, ‘tendon’ and ‘rupture’. Two independent reviewers conducted searches in PubMed, OvidSP for Medline and Embase, as well as the Cochrane Library (Figure 1). Both reviewers triaged articles independently according to the inclusion criteria, and any disputes were settled by a senior author. There was no use of any automation tools throughout the whole process.

Inclusion Criteria

The eligibility criteria included studies on bilateral concomitant patellar tendon ruptures published in English. Bilateral simultaneous patellar tendon ruptures of both traumatic and atraumatic origin in human patients were included. Several articles were excluded for one or more of the following reasons: unilateral patellar tendon rupture, non-simultaneous bilateral tendon rupture, animal study, patellar tendon rupture associated with other knee extensor mechanism injuries (i.e., quadriceps tendon injury) and non-English language (Table 1).

**Table 1:** Total number of studies, number of studies included and excluded, and number of patients and patellas in the included studies; breakdown of included studies according to the type of publication; and reasons for study exclusion. MCL: medial collateral ligament, TKR: total knee replacement

Studies screened		Excluded studies	
Total	183	Duplicate	2
Excluded	138	Language	11
Included	45	Unilateral	6
Patients	45	Quads tendon	50
Patellas	90	Animal study	5
Included	45	MCL	1
Case reports	37	TKR	7
Literature reviews	8	Bone marrow	1

Outcome Measures

The primary outcomes of interest comprised patient factors, diagnostic tools and management options. The secondary outcomes of interest comprised past medical history, Tegner Activity Scale mode of Injury (MOI) (i.e., atraumatic or traumatic), level of injury, bilateral Insall-Salvati ratios, rupture classification, repair technique, intra- and postoperative complications and post-operative management.

Risk of Bias Assessment

We used a risk assessment tool named ROBIS (a tool to assess the risk of bias in systematic reviews), and all the signalling questions were answered. No potential concerns about the review arose.

Results

The literature search identified a total of 183 studies. Following the removal of two duplicates, the remaining 181 studies were analysed according to their title, abstract and full text; 37 studies were excluded, as they were not accessible (the librarian was unable to access most articles before the 1990s). The remaining 144 studies were analysed to assess whether the eligibility criteria were met, which led to the removal of 99 studies and the selection of the remaining 45 studies.

In the final analysis, we included 45 patients and 90 patella tendon ruptures in total. There were no age or demographic restrictions for patients included in the review. We assessed each patient’s past medical history and MOI, the occurrence of direct trauma, the imaging methods used for diagnosis and the types of surgical repair done. Post-operative immobilisation devices and the start of weight bearing (WB) were analysed. Each patella and its corresponding ruptured tendon were classified according to the level of rupture and assessed for post-operative complications and their respective management. The results are presented in table form for ease of reading and clarity.

Out of the 45 patients included in this review, 28 had at least one comorbidity, and out of these, four had multiple comorbidities [9-12]. In those with a history of steroid use, we also looked at the reason behind the use (i.e., treatment or bodybuilding) (Table 2).

**Table 2:** Past medical history analysis.

Past medical history	
Total	45
Fit and well	17
Osgood-Schlatter	1
Steroids	10
Obesity	4
Chronic kidney disease	4
Previous rupture	2
Type 2 diabetes mellitus	2
Ehlers-Danlos syndrome	1
Hypercholesterolaemia	1
Fluoroquinolones	2
Osteogenesis imperfecta	1
Acquired emphysema	1
Hypertension	2
Osteoarthritis	1
Steroids	
Bodybuilding	1
Systemic lupus erythematosus	1
Wegener’s	1
Jumper’s knee	4
Rheumatoid arthritis	2
Pyoderma gangrenosum	1

Patient Factors

In a 2004 systematic review on bilateral concomitant patellar tendon ruptures, the male-to-female patient ratio was 14.5:1, and the median age was 41 years [3]. Regarding the 45 patients included in our review, 41 (91.11%) were male, and four were female (only 8.89%) [13-16], with a mean age of 37.09 years. Comparing the 2004 data with the data we obtained, we can verify that recent literature corroborates that simultaneous bilateral patellar tendon rupture is more common in middle-aged males.

One point of interest in this analysis was determining the relationship between physical activity and rupture injury. We attempted to determine how active the injured patients were and quantify it using the Tegner Activity Scale. However, no conclusions were made due to some limitations. First, a few case reports mentioned the activity level of the patients (28 cases had no information regarding occupation or participation in recreational sports). Second, the application of the Tegner Activity Scale might not have been objective or corresponded to the actual situation due to individual bias and subjective interpretation of the extant text. We calculated a mean score of 6 for the 13 cases in which we applied the Tegner Activity Scale, but we found this information to be of limited significance.

Corticosteroid Use

Of the studies included in our review, 10 patients had a history of steroid use (roughly 22% of the total patients included in the study). Out of those 10 patients, three (33%) had used corticosteroids daily (systemic lupus erythematosus (SLE), Wegener’s and bodybuilding), one had used corticosteroids within the last year but not over the course of the three months leading up to the injury (jumper’s knee), another three (33%) had used corticosteroids over the past three years but not the last year leading to injury (rheumatoid arthritis (RA) and jumper’s knee) and the remaining three (33%) had used corticosteroids more than three years ago (bodybuilding and jumper’s knee).

Corticosteroids are the medications most often linked to tendon rupture due to their anti-mitotic and fibroblast anti-synthetic effects, as well as their stimulation of collagenase. These medications cause the necrosis of fibrils and the disorganisation of collagen arrangement, thus weakening collagenous structures and making them prone to rupture [1].

Disorders of Metabolism

Some patients presented with metabolic disorders, a total of seven patients in the 45 studies (roughly 16%). One patient had a history of hypercholesterolaemia, two presented with a history of type 2 diabetes mellitus (T2DM) and four presented with obesity. The occurrence of simultaneous bilateral patellar tendon rupture in obese patients may be multifactorial, where a sedentary lifestyle may lead to decreased circulation and nutrition of the tendons resulting in degeneration and structural weakness. Furthermore, morbid obesity may lead to a higher level of stress on tendons, joints and bones and generate excessive forces that may lead to severe traumatic orthopaedic injuries resulting from otherwise trivial mechanisms of injury. Kuo and Sonnabend [6] have made reference to McMaster, who proved that the normal patella tendon has considerable tensile strength and usually does not rupture unless weakened by degenerative changes, and to Kannus and Jozsa’s histopathological analysis of 891 specimens from spontaneously ruptured tendons, which revealed evidence of pre-existing pathological changes in all specimens. Zernicke et al. [4] estimated that the force needed to rupture a healthy patellar tendon was 17.5 times one’s body weight.

Hypercholesterolaemia has a well-known impact on vascular and internal organs. Several studies have also explored the relationship between hypercholesterolemia and tendon pathology. Hypercholesterolemia may lead to lipid deposition in the extracellular matrix of the tendon, thus promoting inflammation, structural changes and changes in mechanical properties such as altered stiffness and elastic modulus, which ultimately result in tendon pathology [17-19].

T2DM patients are four times more likely to experience tendinopathy and five times more likely to experience a tendon tear or rupture than non-diabetic patients [20]. Tendon healing among all populations is often unsatisfactory due to the formation of scar tissue rather than the regeneration of the tendon structure; in T2DM patients, this scar-tissue response is amplified, leading to an increased risk of rupture and impaired restoration of range of motion. Structural changes to tendons seen in diabetic tendinopathy, including loss of collagen organisation or thickening and calcification, increase the risk of rupture [20].

Chronic Renal Insufficiency and Hyperuraemic States

The kidney is an essential organ involved in the regulation of calcium and phosphate homeostasis, which is crucial for bone mineralisation and development. Patients with chronic kidney disease often suffer from renal osteopathy, characterised by the metabolic disturbance of bone and minerals. In uraemic patients undergoing long-term haemodialysis, there is often a disturbance of calcium-phosphorus metabolism, which leads to secondary hyperparathyroidism. High levels of parathormone lead to the degeneration of tendon tissue, and a decline in active vitamin D receptors in the parathyroid gland causes a decrease in active vitamin D, which results in ligament and tendon tissue denaturation [21]. Furthermore, metabolic acidosis predisposes one to elastosis, and the biochemical environment of uraemia predisposes one to changes in the protein-polysaccharide complex responsible for the maturation of collagen [16]. Out of the 45 cases analysed in this review, four patients had a history of chronic kidney disease.

Rheumatoid Arthritis

Spontaneous tendon ruptures have been documented in many patients who have rheumatoid arthritis, particularly in the hand and wrist [16]. In our review, we only found two documented cases of bilateral concomitant patellar tendon rupture in patients with rheumatoid arthritis.

Collagenase, related to the inflamed rheumatoid synovium, may be responsible for tendon ruptures in rheumatoid arthritis [22]. Although none of the case reports mentioned any histopathological findings that could corroborate this hypothesis, the case reported by Peiró et al. [23] points out histological findings of avascular changes and fibrinoid degeneration, which are typical in collagen diseases. It is also important to note the possible contribution of steroid therapy as part of the pathophysiological process in the case reported by Wang et al. [24].

Collagen Diseases

Water accounts for 60%-70% of the wet weight of the patellar tendon, and collagen accounts for 70%-80% of its dry weight. The patellar tendon mainly comprises large-diameter collagen fibres (90% are type I collagen, and less than 10% are type III collagen), with elastin, proteoglycans and other non-collagenous glycoproteins forming the remaining tendon matrix [25].

As stated previously, under normal physiological conditions, it is estimated that a force of 17.5 times one’s body weight is required to rupture the patellar tendon [4]. However, a collagen disease will affect the structure of the tendon and make it prone to rupture. Of the 45 patients in our study, two had known collagen disorders: one had osteogenesis imperfecta, and one had Ehlers-Danlos syndrome.

Chronic Microtrauma

It has been hypothesized that only direct trauma can disrupt a healthy patellar tendon [25]. Patellar tendon rupture due to indirect trauma has been considered the end stage of long-standing chronic tendon degeneration secondary to repetitive microtrauma [26] Repetitive microtrauma leads to micro-tearing, which results in tendon degeneration [27]. Four of the cases reported were of patients with jumper’s knee, one of whom had pre-existing osteoarthritis.

Mode and Type of Injury

The mode of injury was a major point of interest in our review, as many of the studies analysed were related to sports activity. We assessed the occurrence of direct trauma, as well as the activity the patients were engaged in at the time of injury. We also collected more information regarding the mechanism of injury when available (Table 3).

**Table 3:** Results of the mode of injury analysis.

Mode of injury	
Total	45
Activity	
Walking	6
Running	1
Sports	18
Fall	20
Mode of injury - detailed	
Sudden quads contraction	20
Hyperflexion	20
Twisting	2
Direct trauma	1
Walking	2
Direct trauma (yes/no)	
Yes	7
Yes (%)	15.56%
No	38
No (%)	84.44%

As previously stated, each injury was classified as one of three types according to the level of rupture of the patellar tendon: type 1, tendon ruptures at the level of its origin at the inferior pole of the patella; type 2, tendon ruptures that occurred at the midsubstance level; and type 3, tendon ruptures that occurred at the level of its insertion at the tibial tubercle (Table 4).

**Table 4:** Type of injury sustained according to the level of tendon rupture. NA: not available

Type/level of injury	
Right	
Type 1	23
Type 2	15
Type 3	3
NA	4
Left	
Type 1	23
Type 2	14
Type 3	4
NA	4
Total	
Total	
Type 1	46
Type 2	29
Type 3	7
NA	8

The Insall-Salvati ratio of each knee was also noted to attempt to determine the mean. However, only eight cases (16 patellas) presented this information (Table 5).

**Table 5:** Mean Insall-Salvati ratio found in left and right knees (37 studies did not present this information). NA: not available

Insall-Salvati ratio			
Right	8	Left	8
Mean	1.62	Mean	1.60
NA	37	NA	37

Investigations and Diagnostic Procedures

We also looked at the investigation methods used (both primary and secondary) to diagnose the ruptures. In one of the cases included in this study, ultrasound and magnetic resonance imaging (MRI) were used as secondary investigation methods (Table 6).

**Table 6:** Investigation methods used to diagnose ruptures. MRI: magnetic resonance imaging, OE: objective examination, USS: ultrasound, NA: not available

Investigation (first)		Investigation (second)	
Total	45	NA	27
X-ray	41	USS	8
OE	3	MRI	11
MRI	1	Count check	46
Count check	45	(1× case USS + MRI)	1

Surgical Management

Every patient in the studies analysed underwent surgical treatment. Surgical repair of the patellar tendon can be done either by direct primary tendon repair or by tendon reconstruction, either with or without augmentation. The results are demonstrated in Tables 7-9.

**Table 7:** Surgical procedures carried out on ruptured tendons.

Surgical procedure	
Total	45
Direct primary repair	
End to end	18
Transosseous	18
Suture anchors	4
Total procedures	40
Tendon reconstruction	
Allograft	0
Auto-gracilis	1
Auto-Achilles	0
Auto-semitendinous	3
Auto-quads	1
Auto-plantaris	1
Total procedures	5
Augmentation	
Procedures with augmentation	23
Procedures with no augmentation	22

**Table 8:** Direct primary repair with augmentation broken down into types of repair and augmentation materials. LARS: Ligament Augmentation Reconstruction System, TBW: tension band wiring

Direct primary repair with augmentation	Allo-LARS	Auto-semi	Auto-Achilles	Auto-gracilis	Cerclage	TBW	Fibertape
End to end	0	2	1	0	6	2	0
Transosseous	1	1	1	1	6	1	0
Suture anchors	0	0	0	0	0	0	2
Total	1	3	2	1	12	3	2

**Table 9:** Tendon reconstruction with augmentation broken down into augmentation materials.

Tendon reconstruction with augmentation		Added cerclage
Allograft	0	0
Auto-gracilis	1	0
Auto-Achilles	0	0
Auto-semitendinous	3	1
Auto-quads	1	1
Auto-plantaris	1	0
Total	6	2

Of all reconstructed tendons, none were repaired using an allograft. The autografts used included the gracilis, Achilles, semitendinous, quadriceps and plantaris tendons. In one patient, autografts from both semitendinous and gracilis tendons were used bilaterally (Tables 8, 9).

Post-operative Management and Complications

Regarding the data collected on the immobilisation device used post-operatively, two patients were immobilised with both a cast and brace, which accounts for the numbers expressed. We also collected data regarding the start of WB, which was only present in 30 of the included studies (Tables 10, 11).

**Table 10:** Post-operative management: immobilisation device used and start of weight bearing (in weeks). NA: not available

Post-operative management	
Immobilisation device	
Brace	25
Cast	13
NA	9
Start of weight bearing (weeks)	
Mean	3.23
NA	15

**Table 11:** Post-operative management: complications and their respective management. DVT: deep vein thrombosis, PE: pulmonary embolism, NA: not available

Post-operative management			
Complications (total)			
Total		14	
Surgical		13	
Anaesthetics		1	
Complications (right)		Complications (left)	
Yes	8	Yes	6
No	37	No	39
NA	0	NA	0
Complications (right) - detailed		Management	
Re-rupture	1	Revision + augmentation (autograft)	
Metalwork break	2	Removal of metalwork	
Anaemia	1	Transfusion	
Irritation	1	Removal of metalwork	
DVT	1	Heparin + coumarin	
PE	1	Heparin + warfarin	
Malignant hyperthermia	1	Tertiary centre referral	
Complications (left) - detailed		Management	
Re-rupture	0	NA	
Metalwork break	1	Removal of metalwork	
Anaemia	1	Transfusion	
Irritation	1	Removal of metalwork	
DVT	0	NA	
PE	1	Heparin + warfarin	
Malignant hyperthermia	1	Tertiary centre - anaesthetics (malignant hyperthermia)	
Chronic infection	1	Adhesiolysis, debridement, biopsies and reconstructions	

Discussion

Disruption of the extensor mechanism of the knee may happen due to various aetiologies, which can be either bone- or tendon-related. These aetiologies comprise an uncommon type of injury, albeit one that requires surgical management to restore physiological function. Patellar fractures are more common than quadricep tendon ruptures, with the latter being more common than patellar tendon ruptures.

The mechanism of injury is common to all injuries to the extensor mechanism of the knee: quadriceps contraction on a flexed knee causing eccentric contraction and subsequent disruption of either tendon or patella. Disruption of the extensor mechanism of the knee typically presents with an inability to extend the knee, palpable local changes at the site of injury and joint hemarthrosis. The non-specific presentation of patellar tendon rupture, the common mechanism of injury compared to other injuries to the extensor mechanism of the knee and its rare occurrence may contribute to its misdiagnosis. Moreover, when occurring bilaterally, a dislodged patella may go unnoticed due to the lack of a healthy knee for comparison. A timely diagnosis is crucial in such injuries, as a lack of or a delay in treatment will lead to serious complications, such as proximal retraction of the patella with scarring, complicated repair and diminished long-term function.

The present systematic review was carried out to collect most of the currently existing data on this injury and derive conclusions via analysis to better understand it, its diagnosis and its treatment, thus improving patient outcomes. As mentioned in the results section, our points of interest included the following: the patient’s medical history, MOI, presence or absence of direct trauma, imaging methods used for the diagnosis, surgical repair techniques and post-operative management (i.e., immobilisation device and start of WB). Each patella and the corresponding ruptured tendon were classified according to the level of rupture and assessed for post-operative complications and their respective management.

Documented risk factors for bilateral patellar tendon rupture include dyslipidaemia, hyperuricemia, tendinopathies, T2DM, fluoroquinolone use, chronic renal insufficiency, haemodialysis, rheumatoid arthritis, collagen disorders and corticotherapy. These factors are responsible for microstructural changes in the ligament substance and the weakening of collagen, thus predisposing the patellar tendon to rupture after minimal trauma or atraumatic situations. The existing literature, in general, broadly divides these risk factors into three major groups: systemic underlying disease, corticosteroid use and chronic microtrauma [17-25]. Of the 45 patients included in this study, only 17 were fit and well, meaning only 37.78% of patients with bilateral patellar tendon rupture had no relevant medical history.

Mode of Injury

Only seven patients of our cohort (15.56%) were injured due to direct trauma [9,28-33], meaning 38 (84.44%) did not have direct trauma accounting for the tendon rupture. Twenty patients sustained their injury as a result of a fall, 18 while doing some sports activity, six while walking and one while running.

Indeed, of the cases reported, 20 mention sudden quadriceps contraction as the mode of injury, and 20 others hyperflexion, which is consistent with the described mechanism of injury to the extensor apparatus of the knee. Two cases mention a twisting motion, and three do not explain the injury further (two cases were sustained while walking, with the remaining cases resulted from direct trauma).

Type of Injury

Regarding patellar tendon rupture, there are three known patterns of injury: avulsion from the inferior pole of the patella, midsubstance and distal avulsion from the tibial tubercle [24-27]. When analysing each ruptured tendon, we classified them according to these patterns as types 1-3, respectively.

We found that 46 of the 90 patellar tendons (51.11%) had type 1 tendon rupture. This finding agrees with the current knowledge that type 1 is the most common patellar tendon rupture type, which can be explained by the fact that strain at the tendon-bone interface is four times higher than that at the midsubstance.

Diagnostic Process

Patellar tendon ruptures are often misdiagnosed [4,13] due to myriad potential causes: the clinical features are not specific to patellar tendon rupture, as previously stated; hematomas are common in acute settings and may conceal some important clinical features; and the rarity and lack of experience with this injury. In cases where patellar tendon rupture occurs bilaterally, having both knees injured may make the dislodged patellas go unnoticed, as there are no means for comparison (i.e., there is no healthy knee to assess the regular physiological position based on clinical examination alone). Therefore, it is important that every adult with knee pain and likely to have a musculoskeletal injury is thoroughly investigated. Proper history-taking and clinical examination alone should raise suspicion for injury of the extensor mechanism of the knee, and imaging methods can confirm the aetiology.

Plain films are an essential diagnostic tool, as they can potentially rule out other associated traumatic pathologies. Specific to patella tendon ruptures, one can find a patella alta (a high-riding patella that is easily identifiable by a high Insall-Salvati ratio of >1.2), the presence of avulsion fractures or the blurring of the posterior edge of the patella tendon into the Hoffa fat pad. When clinically unclear, ultrasonography seems to be a fast, cheap and reliable tool to make this diagnosis; the healthy tendon normally appears as a continuous well-defined hyperechoic fibrillar structure bridging the patella and the tibial tuberosity, whereas tears usually appear as hypoechoic areas of interruption of the fibrillar pattern [34].

Although MRI is considered the gold standard of imaging methods, it seldom provides information that would alter the management of a patella tendon rupture, and it is more expensive and less available than other methods. However, it has been reported [35] that there is a high incidence of intra-articular knee joint injuries in patients with patellar tendon rupture (mostly anterior cruciate ligament and medial meniscus tears); therefore, MRI evaluation should always be considered in young patients.

In our study, we collected data regarding the first and second investigations done in each case. In most cases, the first investigation carried out was an X-ray, which was done in 41 of the 45 cases analysed. Only one case used MRI as the first investigation, and three were diagnosed on just clinical examination.

A second investigation method was not used in 27 of the patients. MRI alone was carried out as a second investigation in 10 patients, and ultrasonography in seven patients. One patient had both MRI and ultrasonography as secondary investigations. Only eight cases [2,24,36-41] reported the Insall-Salvati ratio found during imaging. Of those presented, we found a mean Insall-Salvati ratio of 1.62 for the right patella and 1.60 for the left patella, which confirmed that patella alta is a high-yield finding in the diagnosis of patellar tendon rupture.

Treatment

A therapeutic approach combining prompt surgical repair and physiotherapy is preferred and has shown favourable outcomes. Non-surgical treatment alone is only indicated in partial tendon tears, where the knee extensor mechanism remains intact, and it should be considered in patients who are not surgical candidates due to medical comorbidities. Non-surgical treatment involves immobilising the knee in full extension with a progressive WB exercise program [42].

The surgical treatment modalities for this injury are either direct primary repair or tendon reconstruction. Primary repair is indicated in complete patellar tendon ruptures and in cases where the tendon ends can be approximated. The location of the tear will dictate the type of repair used. The chronicity of the tear is another factor that must be considered. After approximately six weeks, direct repair becomes challenging with native tissue due to tendon retraction. In these cases, choices in treatment are either direct repair with augmentation or tendon reconstruction using autograft or allograft [43-45].

The big majority of the cases reported were treated with direct primary repair (40 patients), and over half of these cases required augmentation of some type, i.e., tendons, cerclage wires or tension band wiring [1-3,11,12,24,28,37,39,40,41,46-54]. The most commonly used method for augmentation was using cerclage wires (12). We found no data in the literature regarding which augmentation method is associated with the best outcomes, and we could draw no conclusions based on this analysis. However, the augmentation method may be an interesting aspect to investigate in the future.

The remaining five patients underwent tendon reconstruction. Although tendon reconstruction with an allograft is possible, all of these patients underwent tendon reconstruction with autografts (either from semitendinosus, quadriceps, plantaris or gracilis tendons). There is plenty of existing literature comparing autograft and allograft use and respective outcomes in anterior cruciate ligament reconstruction, but none comparing autograft and allograft use in patellar tendon reconstruction.

Post-operative Management

Both braces and casts were used as immobilisation devices in the cases reported. No information was present regarding an immobilisation device in nine patients. Braces typically seemed to be more used in the management of these injuries (65.79% of cases; 25 out of 38 patients). Note that two patients wore both casts and braces. Time until the start of WB varied largely (between one and eight weeks), with a mean of 3.23 weeks. However, this information was not present for 15 patients (33% of the cases reported).

Overall, few complications occurred, with 14 knees affected out of 90 (15.56% of repaired knee tendons). Thirteen of these complications were of surgical origin, and one was associated with anaesthetics. These included one case with rupture, three cases with metalwork break, two cases with anaemia, two cases with irritation, one case with deep vein thrombosis, two cases with pulmonary embolism, one case with malignant hyperthermia (which occurred during the first surgery, leading to a delay of the second surgery, pending tertiary centre referral), one case with chronic infection and one case with delayed repair (due to malignant hyperthermia occurrence during the initial surgery).

Upon reviewing the patient data, there were no underlying conditions or apparent risk factors to account for these post-operative complications (except genetic predisposition in the patient with malignant hyperthermia). However, it is interesting to note that both patients whose repairs were complicated by metalwork breakage (occurring bilaterally for one patient and in a single knee for the other) had an early start of WB (after one week). Similarly, the patient whose repair failed and suffered re-rupture of the tendon started WB after two weeks. Although there was insufficient data to derive any conclusions from these results, it could be interesting to try to determine (based on outcomes, rate of recovery and complications) the optimal time for beginning WB after patellar tendon rupture repair [45]. Upon review of the reported cases, one can confidently say that surgical repair of the bilateral patellar tendon has good outcomes.

Strengths and limitations

The pathophysiology of tendon ruptures is well documented in medical literature as well as all its exacerbating factors. Bilateral concomitant injuries, although much less prevalent than unilateral injuries, show similar risk factors.

Although we were able to extensively describe the pathophysiology behind each risk factor, one important data point that most studies lacked to provide was an objective functional assessment of the patient pre- and post-injury. Such data would possibly allow us to better comprehend the relevance of each risk factor and its impact on post-surgery functional outcomes.

Future research

This systematic review also revealed gaps in the literature, and further high-evidence studies are needed to improve care for these patients and evaluate their outcomes, particularly in terms of pre- and post-injury functional assessments.

## Conclusions

Bilateral patellar tendon rupture is a rare injury that should be considered in every patient with apparent knee extensor mechanism disruption, despite past medical history. Because examination signs are non-specific, an X-ray of each knee should be considered in anyone presenting with knee pain and swelling, as it is usually sufficient for diagnosis. Surgical treatment and physiotherapy generally have good outcomes with a low rate of complications.
